# Child and Adult Factors Related to Quality of Life in Adults with Autism

**DOI:** 10.1007/s10803-017-3105-5

**Published:** 2017-03-25

**Authors:** Philippa Moss, William Mandy, Patricia Howlin

**Affiliations:** 1 Great Ormond Street Hospital, Level 3, Italian Building, Great Ormond Street, London, WC1N 3JH UK; 2 Research Department of Clinical, Educational and Health Psychology, University College London, Gower Street, London, WC1E 6BT UK; 3 Institute of Psychiatry, Psychology and Neuroscience, King’s College, Denmark Hill, London, SE58AF UK; 4 Faculty of Health Sciences, Brain & Mind Centre, University of Sydney, Camperdown, NSW 2050 Australia

**Keywords:** Autism, Quality of Life, Adult, Adult outcome

## Abstract

The WHO Quality of Life-Brief questionnaire was used to assess quality of life (QoL) among 52 adults with autism (mean age 49 years) followed-up since childhood. Overall, assessments of QOL were more positive than measures of objective social outcome (jobs, independence, relationships etc.) but correlations between caregiver and self-reports were low. Informant ratings indicated few correlations between current QoL and any child or adult factors. On self-report ratings, QoL was significantly negatively correlated with severity of repetitive behaviours in childhood; higher QoL was positively associated with better adult social outcomes. However, only a minority of adults (n = 22) could provide self-report data and findings highlight the need to develop valid measures for assessing the well-being of adults with autism.

**Electronic supplementary material:**

The online version of this article (doi:10.1007/s10803-017-3105-5) contains supplementary material, which is available to authorized users.

## Introduction

Most studies of adults with autism spectrum disorders (ASD) suggest that prognosis, as assessed by objective measures of social outcome (e.g. independence, employment, social relationships), is poor (Howlin and Magiati 2017). However, recently there been a focus on broader and more subjective measures, such as those assessing general quality of life (QoL). Although quality of life in autism tends to be lower than in the general population (Barneveld et al. 2014; Chiang and Wineman 2014; Egilson et al. 2016; Ikeda et al. 2014; Jonsson et al. 2016; Kamp-Becker et al. 2011; Van Heijst and Geurts 2015), assessments of QoL amongst adults with ASD often prove more positive than normative measures of social functioning (Billstedt et al. 2011; Bishop-Fitzpatrick et al. 2016; Helles et al. 2016; Henninger and Taylor 2013; Hong et al. 2016; Renty and Roeyers 2006). Nevertheless, findings vary according to the particular QoL measures and methodologies used (Jonsson et al. 2016). Several studies, mainly involving children and adolescents, have also reported significant discrepancies between self and informant ratings (Clark et al. 2015; Ikeda et al. 2014; Jonsson et al. 2016). Although self-reports of QoL tend to be more positive than informant reports (e.g. Clark et al. 2015; Egilson et al. 2016; Hong et al. 2016; Ikeda et al. 2014) this is not always the case (Jonsson et al. 2016). Moreover, findings on the associations between QoL and factors such as autism severity, age, gender, cognitive, social and language skills, adaptive behaviours, behavioural disturbance, physical health, and co-morbid psychiatric conditions are variable, and sometimes contradictory (Biggs and Carter 2016; Chiang and Wineman 2014; Hong et al. 2016; Ikeda et al. 2014; Kamio et al. 2013; Van Heijst and Geurts 2015). Suggestions that support networks may have a stronger impact on QoL in autism than individual characteristics (Renty and Roeyers 2006) have also led to exploration of a wide range of potential environmental influences. These include bullying, maternal warmth and relationships, participation in social and recreational activities, level of school inclusion, quality of neighbourhood, independence in daily activities, and levels of stress (Bishop-Fitzpatrick et al. 2016; Hong et al. 2016; Woodman et al. 2016). However, the relative influence of these variables, the interactions between them, or how associations may change with age, remains uncertain.

### Background to the Present Study

Over the past four decades years we have followed up a cohort of 60 individuals initially diagnosed with autism as children (see Howlin et al. 2004 for details). Child diagnoses (at mean age 6 years) were confirmed with the Autism Diagnostic Interview (Le Couteur et al. 1989) and re-confirmed in adulthood (mean age 44 years) using the Autism Diagnostic Interview-Revised (ADI-R; Rutter et al. 2003). As children, all participants had a non-verbal IQ of ≥70; data were also collected on language and autism severity (Howlin et al. 2004). Adult data included assessments of cognitive and social functioning, and mental health. Almost all individuals (92%) showed a decrease in autism severity over time and IQ, for most, remained stable or increased (Howlin et al. 2013, 2014; Moss et al. 2015). Nevertheless, most adults (60%) were rated as having a poor social outcome and 28% had severe mental health problems. Outcome was particularly poor in those individuals (25% of sample) who, for behavioural or other reasons, could no longer complete standard IQ assessments and therefore showed deterioration in measured IQ (Details in Supplementary Material, note 1; Howlin et al. 2014). Information collected on this cohort over many years allowed us to examine the association between individual characteristics (both child and adult) and current quality of life.

### Study Aims

Our principal research aims were:

1. To assess quality of life in a UK cohort of adults with autism, using the WHO Quality of Life-Brief Version (WHOQOL-BREF, 1998; see below).
2. To investigate the association between self and informant perceived QoL scores in those individuals who were able independently to complete the WHOQOL-BREF.
3. To examine childhood and adult characteristics associated with current QoL. Variables studied included age, autism severity, IQ and language, and, in adulthood, ratings of social outcome and mental health. We also explored whether there was any association between current QoL and *changes* in IQ or autism symptomatology scores over time. Given the inconsistencies in previous research findings, no specific hypotheses about the relationship between QoL and the variables studied were proposed.

## Method

### Recruitment Procedure and Ethics

Of 60 families involved in the adult follow-up cohort (Howlin et al. 2013, 2014), 59 had consented to be contacted for future research. Approximately 3–4 years after their previous assessments, letters requesting participation in a study of quality of life were sent to these families and included an information sheet, consent forms, the WHOQOL-BREF informant version, and, if appropriate, the self-report version (see below). In total, quality of life data were available for 52 individuals (43 male, 9 female; positive response rate: 88% from previous adult follow-up). Non-responders (n = 7) were similar to responders in terms of age, IQ and mental health but they had lower autism severity scores (d = 1.08) and better social outcome ratings (d = 0.63) than responders (Supplementary Table 1).

Ethical approval was granted by the Maudsley hospital/Institute of Psychiatry and UCL ethics committees (project references IOP 07/H0807/65; UCL 4111/001). Informed consent was obtained from all participants.

### Participants

Data on IQ, language and autism severity were collected in childhood (*M* = 6.3 years; *SD* = 2.1, range 2–13 years) and in adulthood (*M* = 47.9 years; *SD* = 9.5; range 33–68 years). In adulthood, information was also gathered on social functioning and mental health (see Tables 1, 2; full details:Howlin et al. 2004, 2013, 2014; Moss et al. 2015).

**Table 1:** Child and adult characteristics

	Child data	Adult data	t
	Mean (SD, range)	Mean (SD, range)	
Best estimate IQ^a^	89.3 (14.4, 70–133)	69.9 (32.4, 20–139)	−4.12***
ADI/R scores			
Total	40.1 (7.4, 25–56)	27.6 (7.8, 7–47)	−11.54***
Reciprocal social interaction	19.0 (5.3, 5–28)	13.8 (4.7, 2–20)	−7.49***
Communication	13.9 (3.7, 6–22)	10.0 (4.1, 2–20)	−5.96***
RRBI	7.4 (2.2, 2–11)	3.7 (2.2, 0–9)	−10.87***

Language level	N^2^ (%)	N (%)	Χ^b^
Good	26 (51%)	39 (75%)	5.39*
Few words	11 (22%)	8 (15%)	
None	14 (27%)	5 (10%)	

^a^Best estimate 1Q based on highest level IQ test that participant was able to complete (See Supplementary Note 2)

^b^Language score missing for one person. *p ≤ 0.05; ***p ≤ .001

**Table 2:** Adult participant ratings: Composite ratings for social outcomes and mental health

	*N (%)*
Adult social outcome rating	
Very good/good	5 (10%)
Fair	15 (29%)
Poor/very poor	32 (62%)
Mental health rating^a^	
Good/very good	31 (63%)
Moderate	1 (2%)
Poor/very poor	17 (34%)

See Supplementary Tables 3 (a & b) and 4 for details of codings

^a^Mental health outcome scores missing for three participants

The informant WHOQOL-BREF was completed by 50 caregivers (37 parents, 7 siblings; 6 care staff). There was no effect of informant on QoL ratings (See Supplementary Note 2). The self-report version was completed by 22 adults. This relatively low number is due to the fact that several participants were unable to complete standardised assessment measures at follow-up (See Supplementary Note 1; Howlin et al. 2014). Thus, only individuals able to complete a standardised IQ test were asked to complete the self -report measure. As well as these individuals being more cognitively able than the rest of the cohort they had significantly fewer autism symptoms and higher social outcome ratings (Supplementary Table 2).

### Measures

#### Adult Assessments

##### Quality of Life

The WHOQOL-BREF (WHOQOL Group, 1998) is a 26-item questionnaire based on the original 100-item WHOQOL. Ratings (0-*Very poor to 4-Very good*) are based on the past month and generate four domain scores: (i) Physical health, (ii) Psychological well-being, (iii) Social relationships, (iv) Quality of environment. Scores are transformed into a 0–100 scale. Mean scores for a UK “Well” sample (n = 1324) range from 67 to 76 for individual domains (Skevington and McCrate 2012). The measure also includes two general questions; (Q1) ‘How would you rate [his/her][your] quality of life?’ and (Q2) ‘How satisfied [are you] [is he/she] with [your] [his/her] health?’.

The WHOQOL-BREF has good-to-excellent psychometric properties (Hawthorne et al. 2006; Skevington and McCrate 2012) and has been used with a range of samples including a higher ability adult autism group (Kamio et al. 2013). As most (62%) of the adults in the present study had been living away from home for some years and 25% had no or very limited phrase speech, informants’ own perceptions of the adult’s quality of life were assessed rather than proxy ratings (i.e. carers reporting what they think the individual with ASD believes their own QoL to be).

##### Autism Symptomatology, Cognitive and Language Level

Autism severity was assessed using the “Current” form of the ADI-R (Rutter et al. 2003) completed by parents/caregivers. IQ scores were based, where possible, on the Wechsler Adult Intelligence Scale-III (Wechsler et al. 1997). If an individual could not complete this, an alternative test was used (Supplementary Note 3). Language was rated using the ADI/ADI-R summary categories (<5 words; no functional phrase speech; functional use of phrase speech).

##### Social and Mental Health Outcomes

These were assessed using the informant version of the Family History Schedule (FHS), a semi-structured interview used in many autism studies (e.g. Bolton et al. 1994; Pickles et al. 2000; Pinto et al. 2010). A composite social outcome score was derived from ratings for employment, relationships and independent living. A composite mental health score was based on FHS scores for five areas of mental health difficulties (OCD, episodic depression, chronic depression, bipolar disorder, anxiety disorder). (See Supplementary Tables 3 (a & b) and 4; Howlin et al. 2013; Moss et al. 2015).

#### Childhood Data

Child Performance IQ scores were obtained from the test most appropriate for the child’s mental age. Autism severity scores and ratings of language level at diagnostic confirmation were based on the ADI (Le Couteur et al. 1989). (Full details in Howlin et al. 2004, 2013, 2014).

### Data Analysis

Power analysis was based on earlier follow-up research (Farley et al. 2009; Howlin et al. 2013) reporting significant associations between adult outcome and various child and adult variables (range −0.34 to 0.83). Using these data, and assuming a correlation ≥0.4 is of likely clinical significance (Pallant 2007), a sample size of 47 was required for a Pearson correlation to have 80% power to detect a statistically significant association (two-tailed alpha = 0.05).

Parametric tests were used unless assumptions of normality were violated. Due to the number of group comparisons conducted, significance level was set at *p* < .01 (all tests 2 tailed); effect sizes (Cohen’s d/Hedges delta) are also reported. Given the exploratory nature of the correlational analyses, all findings with a *p* value <0.05 are discussed. Regression analysis was conducted only if there were multiple significant correlations with a particular QoL domain.

## Results

Table 3 summarises WHOQOL-BREF informant and self-report scores for the total sample. On informant ratings, the proportions scoring within at least one standard deviation of population norms (Skevington and McCrate 2012) were as follows: Physical 89%; Psychological 78%; Social 80%; Environment 98%. For self-ratings the proportions within one standard deviation of population norms were: Physical 100%; Psychological 91%; Social 91%%; Environment 100%.

**Table 3:** WHOQOL-BREF scores: self-report and informant ratings (all participants)

Ratings	All autism participants	All informants	t	d/δ
	Mean (SD) [n^a^]	Mean (SD) [n^a^]		
Physical	81.1 (9.9) [22]	72.4 (13.7) [47]	2.66**	.73
Psychological	72.1 (15.8) [22]	63.2 (13.2) [45]	2.42*	.61
Social	69.5 (23.3) [20]	56.1 (14.7) [45]	2.81**	.57
Environment	76.6 (11.0) [22]	74.4 (10.3) [49]	0.82	.21
Q1	4.1 (0.71) [22]	4.2 (0.69) [48]	0.56	.15
Q2	3.8 (0.92) [22]	3.4(0.73) [47]	1.48	.43

^a^Not all individuals completed each domain. *p ≤ .05; **p ≤ .01

For the sample as whole, informant ratings for the physical, psychological and social domains tended to be lower than self-ratings. Despite the higher cognitive and social functioning of individuals who were able to self-report (Details Supplementary Table 2) there were no differences in informant ratings for participants with or without self-report data (Table 4); however, effect sizes for the Psychological domain were in the moderate range.

**Table 4:** WHOQOL-BREF ratings: comparison of informant scores for individuals with and without self-report data

	Informant ratings for individuals without self-report data	Informant ratings for individuals with self-report data	t^b^	d/δ
	Mean (SD) [n^a^]	Mean (SD) [n^a^]		
Physical	69.8 (15.8) [27]	75.9 (9.4) [20]	1.53	.39
Psychological	59.9 (13.7) [26]	67.6 (11.5) [19]	1.97	.61
Environment	74.8 (12.2) [29]	73.8 (6.8) [20]	0.33	.08
Q1	4.2 (0.83) [29]	4.2 (0.41) [19]	0.15	.00
Q2	3.3 (0.76) [29]	3.5 (0.71) [18]	0.86	.27

^a^Not all individuals completed each domain

^b^No *p* values <0.05

Comparisons of self and informant scores for those individuals (n = 20) with both sources of data (Table 5) indicated that self-report scores were generally higher but the difference only reached significance, with moderate effect sizes, for the social domain and Question 1 (overall Quality of Life). Although most self-informant correlations were small to moderate, agreement on the social and environmental domains was extremely low.

**Table 5:** WHOQOL-BREF: Informant and participant scores for those individuals (n = 20) with both sources of data

QoL domain	Informant	Autism participants	r	t	d
	Mean (SD)	Mean (SD)			
Physical	75.9 (9.4)	80.8 (10.3)	.35	−1.92	49
Psychological^a^	67.6 (11.5)	72.8 (17.1)	.39	−1.38	.36
Social	59.1 (8.5)	71.3 (23.4)	.01	(z^b^ = −1.96)	.69
Environment	73.8 (6.8)	76.8 (11.5)	.02	−.91	.32
Q1	3.9 (0.9)	4.4 (0.8)	.47*	−2.44*	.59
Q2	4.0 (1.0)	4.3 (0.7)	.23	−1.55	.35

^a^Psychological ratings missing for one pair. *p ≤ 0.05.; ^b^Wilcoxon z used as data significantly skewed

### Variables Associated with Adult QoL (See Supplementary Tables 5 & 6 for all correlations)

#### Childhood Factors

On the informant WHOQOL-BREF there were no significant correlations with any childhood factors. On the self-report measure, there was a significant negative correlation between ADI total score and overall satisfaction with health (Q2) (*r* = −0.55, *p* < .01; i.e. greater autism symptom severity is associated with less satisfaction with health). Poorer overall self-reported QoL (Q1) was associated with higher childhood IQ (*r* = −0.44); higher levels of childhood repetitive and stereotyped behaviours were associated with poorer physical (*r* = −0.44), psychological (*r* = −0.50) and environmental (*r* = −0.51) QoL (all *p* values <0.05).

#### Adult Factors

On the Informant measure, only one association emerged, between older participant age and poorer physical QoL (*r* = −0.34; *p* < .05). On the self-report WHOQOL, social satisfaction scores were significantly positively associated with adult social outcome ratings (*r* = 0.57; *p* < .01) but negatively associated with IQ (*r* = −0.56; *p* < .05). When these two variables were entered in a regression model, the overall model was highly significant [F(2,16) = 8.4, *p* = .003), explaining a large proportion of variance in social QoL (r^2^ = 0.51). Within this model social outcome was a significant predictor (standardised beta = 0.54, *p* = .024); adult IQ was no longer significant (standardised beta = −0.24, *p* = .286).

#### QoL and Changes in Cognitive Ability and Autism Symptoms over Time

There was no significant association between change in ADI scores over time and current QoL total score, (self [r = 0.11] or informant [r = 0.16]). The correlation with change in IQ was non-significant for self-report QoL (r = 0.03) but just reached significance for informant QoL (r = 0.35; *p* < .05). Further analysis indicated no significant group differences (at *p* < .01) in any informant-based QoL ratings between individuals showing a significant decline (>2 standard deviations) in cognitive scores and individuals whose IQ had remained stable from child to adulthood. (Details Supplementary Table 7).

## Discussion

The present study investigated informant perceived and self-report ratings of quality of life among a cohort of adults with autism first seen in early childhood. Replicating the findings of Hong et al. (2016), most adults and informants reported relatively good QoL. WHOQOL-BREF ratings (self and informant) were also comparable to those reported by Hong et al. (2016) and were mostly within one standard deviation of the means reported for individuals without disabilities in the general population (Hawthorne et al. 2006; Skevington and McCrate 2012; see Supplementary Table 8). Overall, findings are in agreement with previous studies suggesting that measures focusing on general well-being provide a more positive picture than those focussing on outcomes such as jobs and independent living (e.g. Billstedt et al. 2011; Bishop-Fitzpatrick et al. 2016; Helles et al. 2016; Henninger and Taylor 2013; Hong et al. 2016; Renty and Roeyers 2006).

Nevertheless, correlations between self and informant reports were generally low. In particular, self-ratings for social relationships were significantly more positive than informant scores, suggesting that adults in this sample were more satisfied with their social lives than perceived by others. Hong et al. (2016) also found that self and maternal ratings were generally similar, apart for social relationships, which, as in the present study, were viewed more positively by the individuals with ASD.

Existing data on the individual characteristics correlated with QoL are often inconsistent, with different studies identifying very different associations. Unlike some other studies (e.g., Bishop-Fitzpatrick et al. 2016; Helles et al. 2016; Kamp-Becker et al. 2011), we identified few child or adult variables that were strongly associated with current self or informant rated QoL. Similarly, there was little association between WHOQOL-BREF scores and changes in IQ or autism symptomatology over time. Other variables that might be expected to show some degree of association (e.g. ratings of adult social functioning and WHOQOL-BREF social domain informant scores; adult mental health ratings and WHOQOL-BREF psychological well-being) showed no relationship.

Self-reports indicated rather more, albeit moderate, associations between childhood variables and adult QoL than informant reports. In particular, higher self-ratings on the social satisfaction domain were related to more positive social outcome scores. There were significant negative associations, too, between WHOQOL-BREF self-report and severity of childhood autism features, especially related to stereotyped and repetitive behaviours. This parallels the reported association between higher levels of early autistic symptomatology and lower, objective ratings of adult outcome (Howlin and Magiati 2017). Although previous research has suggested that higher adult IQ is associated with better social outcomes (Howlin and Magiati 2017), in this cohort individuals with higher cognitive levels tended to report lower social satisfaction. However, the effects of IQ were limited when overall social functioning was taken into account.

### Strengths and Limitations

The present study has a number of strengths. These include the relatively large sample size, the high retention rate over the years, and access to a range of child and adult measures permitting analyses of factors related to current quality of life. Nevertheless, various methodological issues limit the generalizability of findings. In particular, this was a highly selected cohort, diagnosed at a time when autism was far less well recognised and hence participants may have had more severe autistic symptomatology than is typical of children of average IQ who are currently diagnosed. Moreover, only a minority of adults completed the self-report measure and statistical power for those analyses was low. Individuals able to self-report were also more able than those for whom only informant data were available, although informant ratings for these two subgroups were similar. In addition, we have no information about QoL among those individuals (n = 7) who did not participate in this phase of the follow-up study. It is also unknown whether face-to-face administration of the WHOQOL-BREF, possibly using a modified format (cf. Hong et al. 2016), might have affected the results and/or allowed more adults to take part. It is possible, too, that agreement between self and informant data may have been greater if proxy reports had been used instead of relying on caregivers’ perceptions of individuals’ QoL (Hong et al. 2016; Sheldrick et al. 2012). Correlations between the WHOQOL-BREF and other child variables may also have been attenuated by the relative homogeneity of IQ in childhood; however, that would not explain the lack of correlations with IQ in adulthood, where the range was wider. A further caveat is the lack of information on environmental variables (family factors, specific interventions, educational and social provision etc.) that have been identified as important in other studies (e.g. Bishop-Fitzpatrick et al. 2016; Renty and Roeyers 2006). Finally, there was a 3–5 year gap between the assessments of IQ and social functioning/mental health and the collection of WHOQOL-BREF data, although there was no evidence of any significant changes in psychological, physical or social circumstances during this time.

### Measuring Quality of Life

Despite increasing focus on quality of life for adults with autism, there is little agreement on the most appropriate measures to use, or which of the multitude of variables that might affect QoL should be studied. Correlations between self and informant reports are typically low and data on variables associated with QoL are often inconsistent. The inability (or unwillingness, cf Helles et al. 2016) of some individuals to complete standard QoL assessments also raises questions about the utility of such measures for the wider autism population, especially for individuals of lower ability. The validity of proxy reports (what the caregiver thinks the individual with ASD believes his/her own QoL to be) remains uncertain for adults with very low cognitive and communication skills. On the other hand, informant perceived QoL (caregivers’ own perceptions of the adult’s quality of life) might be affected by their own aspirations and/or anxieties for his/her future. And, even for more able individuals with autism, we cannot be certain that they interpret self-report questions in the same way as in the general population.

In the present study, WHOQOL-BREF scores were comparable to those reported for another adult cohort of slightly younger but more intellectually impaired adults (Hong et al. 2016). Informant scores for higher and lower ability participants were also very similar, suggesting the potential utility of this instrument across a relatively wide range of ability. Nevertheless, only a minority of participants was able to self-report, and the impact of some suggested adaptations (to wording, content, mode of presentation, or scoring; cf. Hare et al. 2015; Hong et al. 2016; Power and Green 2010) to increase participation, remains unknown. Instead, Tavernor et al. (2013) recommend the development of a syndrome specific measure of QoL, constructed with the active involvement of individuals with autism and their families.

## Conclusions

Although previous research on autism in adulthood indicates that prognosis for most individuals is poor, studies focussing on more general measures of wellbeing indicate a more encouraging outlook. However, conclusions differ according to the information source and measures used, and many questions remain about the factors, both individual and environmental, that influence quality of life. Given growing demands for autism research to reflect the values of individuals with autism themselves (Pellicano et al. 2014), it is becoming increasingly important to develop appropriate, valid and comprehensive measures of wellbeing that can be used across the whole autism spectrum.

## Electronic supplementary material

Below is the link to the electronic supplementary material.

Supplementary material 1 (DOCX 58 KB)
