# Supplementary material for: Child and Adult Factors Related to Quality of Life in Adults with Autism
Source: J Autism Dev Disord. 2017 Mar 25;47(6):1830–7. doi: 10.1007/s10803-017-3105-5 (PMC5432629; doi:10.1007/s10803-017-3105-5)
Supplement: Supplementary file 1 — Supplementary material 1 (DOCX 58 KB) [file 10803_2017_3105_MOESM1_ESM.docx]

**ON LINE SUPPLEMENTARY INFORMATION**

**Note 1. Individuals no longer able to complete cognitive assessments in adulthood.**

In 75% of the original cohort who were able to complete at least a non-verbal IQ test in adulthood, non-verbal IQ remained very stable from child to adulthood and verbal IQ significantly increased.

|  |  |  | *t* |
| --- | --- | --- | --- |
| NON-V IQ | 88.5 (14.1)  *[60]* | 89.8 (21.8)  *[44]* | *-0.5* |
| VIQ | 79.6 (23.2)  *[23]* | 85.6 (22.4)  *[33]* | *-2.0** |

However, 15 individuals were unable to complete standardised IQ tests as adults and for them follow-up cognitive estimates were based on Vineland Adaptive Behavior Scale scores. Regression analysis indicated that age at initial assessment was the strongest predictor of decline in change in IQ followed by language in childhood (younger age at initial assessment and poor language ability in childhood = >decline in IQ). There were no correlations with gender, current age, child IQ, or diagnostic ADI scores. Participants with epilepsy also had significantly lower IQ scores in adulthood than those who did not (p=0.01). In most cases the apparent decline in cognitive ability was reported to have occurred in adolescence/early adulthood and was associated with the onset of persisting behaviour problems that affected their ability to complete IQ assessments as adults

**Note 2.**

**Maternal rated vs non maternal** reported scores were compared (using t tests or Mann Whtiney tests as appropriate) for each WHOQOL sub scale, the total score and the QoL summary items (Q1 and Q2). There was no evidence for any effect of informant on WHOQOL score - all comparisons: p>.13

**Note 3. Cognitive assessments**

Because of the variable abilities of the participants and differences in IQ tests for children and adults, assessment measures varied over time. A “Best Estimate” IQ score was derived for each participant based on the best quality, age appropriate test completed. This was designed to give the best estimate, from all the assessments available, of an individual’s general level of cognitive ability. For the sample as a whole, Best Estimate scores in childhood were based on the WISC/WISC-R or WPPSI (Wechsler, 1949; 1974; 1967) Merrill Palmer ([Stutsman, 1948](#_ENREF_221)) or Leiter scales (Levine and Leiter, 1982). At final adult assessment Best Estimate IQ was based mainly on the WAIS-III (Wechsler, 1997); the WASI (Full scale; Wechsler, 1999) was used for 9 individuals who were reported to have difficulties with longer assessments. Raven’s Coloured Matrices scores were used as a proxy for Best Estimate IQ for ten adults with very limited speech. Fifteen adults did not complete any direct assessments, either because they clearly could not understand test instructions or requirements and/or because of behavioural problems such as aggression or self-injury which their carers feared would be exacerbated by attempts to test them formally. For them a “Best Estimate” score was based on their developmental level as assessed by the Vineland Adaptive Behavior Scales (VABS; Sparrow, Cicchetti and Balla, 2005). One participant refused any psychometric testing at follow-up because of disapproval of normed assessments.

**Supplementary Table 1: Adult characteristics of non-responders (n=7) and responders (n=52)**

|  | **Non-responders** | **Responders** |  |  |
| --- | --- | --- | --- | --- |
|  | **Mean (SD, range)** | **Mean (SD, range)** | **t** | **d/δ** |
| **Age** | 44.7  (7.9) | 49.3  (9.5) | 1.21 | 0.52 |
| **Best Estimate IQ^1^** | 79.3  (42.9) | 69.9  (32.4) | .69 | 0.24 |
| **ADI /R scores**  Total | 18.8  (8.4) | 27.6  (7.8) | 2.59* | 1.08 |
| Reciprocal Social Interaction | 9.8  (6.8) | 13.8  (4.7) | 1.88 | 0.70 |
| Communication | 6.7  (3.0) | 10.04  (4.1) | 1.97 | 0.93 |
| RRBI | 2.0  (1.4) | 3.7  (2.2) | 1.84 | 0.92 |
| **Social outcome rating** | 6.5  (4.9) | 9.0  (3.2) | 1.85 | 0.63 |
| **Mental health rating** | 0.67  (1.2) | 1.2  (1.6) | 0.79 | 0.37 |

^1.^ Best estimate 1Q based on highest level IQ test that participant was able to complete (See Supplementary Note 2);

*p≤.05

**Supplementary Table 2: Adult characteristics of individuals with/without self report data.**

|  | **Individuals with self-report data (n=22)** | **Individuals with informant data only (n=30)** |  |  | |
| --- | --- | --- | --- | --- | --- |
|  | **Mean (SD, range)** | **Mean (SD, range)** | **t** | **d/δ** |  |
| **Age** | 51.3  (9.2) | 47.8  (9.6) | 1.31 | 0.37 |  |
| **Best Estimate IQ^1^** | 91.4  (19.1) | 54.9  (31.5) | 4.72*** | 1.40 |  |
| **ADI /R scores**  Total | 22.7  (7.2) | 31.1  (6.3) | 4.45*** | 1.25 |  |
| Reciprocal Social Interaction | 10.9  (4.3) | 15.9  (3.8) | 4.46*** | 1.23 |  |
| Communication | 8.9  (2.5~~)~~ | 10.8  (4.7) | 1.63 | 0.50 |  |
| RRBI | 2.9  (1.9) | 4.3  (2.1) | 2.38** | 0.69 |  |
| **Social outcome rating** | 6.9  (2.9) | 10.6  (2.5) | 4.82*** | 1.37 |  |
| **Mental health rating** | 1.3  (1.8) | 1.2  (1.5) | 0.28 | 0.06 |  |

^1.^ Best estimate 1Q based on highest level IQ test that participant was able to complete (See Supplementary Note 2)

. p*≤.05;**p≤.01; ***p≤.001

**Supplementary Table 3a. Social Outcome Ratings: Independence, employment, friends and relationships.**

| **Social Domains** | **Descriptors** | **Score** |
| --- | --- | --- |
| Independence | Living Independently | 0 |
|  | In semi-sheltered accommodation (or still at home) but with high degree of autonomy | 1 |
|  | Living with parents, some limited autonomy/In residential accommodation with some limited autonomy | 2 |
|  | In specialist autistic or other residential accommodation with little or no autonomy/ In hospital care or at home because nowhere else available | 3 |
| Friendships | 1/+ relationships, in approximately own age group | 0 |
|  | 1/+ relationships but limited in terms of restricted interests or less than normal reciprocity | 1 |
|  | Seeking of contact but only in group situation/school/work | 2 |
|  | No peer relationships that involves selectivity | 3 |
| Highest Level of Employment | Professional or highly skilled and non-manual skilled jobs | 0 |
|  | Manual skilled | 1 |
|  | Partly skilled or unskilled and untrained | 2 |
|  | Chronically unemployed, year, sheltered employment, never worked | 3 |
| Current Employment | Employed or self-employed | 0 |
|  | Out of work up to 5 years | 1 |
|  | Out of work 5 years+ | 2 |
|  | Never had a job | 3 |
| Highest Level of Relationships | Has maintained reciprocal relationships | 0 |
|  | Reciprocal relationships but typically shorter than normal for peer group | 1 |
|  | No enduring relationships/relationships all very brief with reduced sharing | 2 |
|  | No reciprocal relationships longer than one month/never had a relationship | 3 |

**Supplementary Table 3b. Composite Social Outcome Ratings**

| **Description** |  | | **Score Overall rating** | |
| --- | --- | --- | --- | --- |
| Achieving a high level of independence, having some friends and is employed / highly professional / non-manual skilled; maintained reciprocal relationships | | | 0-2 | 0-Very Good |
| Requiring some degree of support in daily living; employed/out of work up to 5 years; professional/manual skilled; some friends/acquaintances; has experienced relationships but typically shorter than normal | | | 3-5 | 1-Good |
| Has some degree of independence, and although requires support and supervision does not need specialist residential provision; out of work up to and over 5 years; manual skilled/partly skilled; no close friends but some acquaintances; reciprocal relationships with reduced sharing | | 6-8 | | 2-Fair |
| Requiring special residential provision/ high level of support; partly skilled/ never had a job; no friends outside of residence; no enduring relationships | | 9-11 | | 3-Poor |
| Needing high-level hospital care, no friends; no autonomy; never had a job; no relationships | | 12+ | | 4-Very Poor |

**Supplementary Table 4. Mental Health Ratings**

***Scoring for individual disorders***

0 = none

1 = symptoms lasted < 1 month, or no significant distress or impairment

2 = symptoms lasting ≥1 month, associated with distress or impairment and/or received treatment

3 = hospitalised for at least 2 days, or required ECT.

| **Overall mental health rating** |  | |
| --- | --- | --- |
| **Very good** | | No difficulties in any area (all items scored 0) |
| **Good** | | Mild symptoms (i.e. score of 1) in one area |
| **Moderate** | | Mild difficulties (score of 1) in >1area |
| **Poor** | | Persistent problems and/or requiring treatment (score of 2) in 1 area |
| **Very Poor** | | Severe difficulties (score of 2) in >1 area  *Or* In-patient hospital treatment required.  *Or* Severe difficulties in one area and mild difficulties in 2 areas |

**Supplementary Table 5. Correlations between adult QoL and childhood factors**

| ***Domain*** |  | ***Correlations*** | | | ***Diagnostic ADI algorithm*** | | |
| --- | --- | --- | --- | --- | --- | --- | --- |
|  | ***n*** | ***IQ*** | ***RSI*** | ***Communication*** | | ***RRBI*** | ***Total*** |
| ***Informant*** |  |  |  |  | |  |  |
| *Physical* | 47 | .02 | .05 | -.04 | | .11 | .00 |
| *Psychological* | 45 | -.12 | -.03 | .14 | | .12 | .01 |
| *Social* | 45 | -.04 | .28 | -.11 | | .23 | .19 |
| *Environment* | 49 | -.05 | .14 | -.07 | | -.05 | .03 |
| *Q1* | 50 | -.20 | -.08 | -.16 | | .02 | -.16 |
| *Q2* | 50 | -.16 | .00 | -.09 | | -0.09 | -.10 |
| ***Self-report*** |  |  |  |  | |  |  |
| *Physical* | 22 | -.34 | .14 | -.02 | | -.44* | -.10 |
| *Psychological* | 22 | -.26 | .21 | -.07 | | -.50* | -.05 |
| *Social* | 20 | -.36 | .36 | .19 | | -.24 | .22 |
| *Environment* | 22 | .04 | -.03 | -.12 | | -.51* | -.16 |
| *Q1* | 22 | -.44* | .02 | .06 | | -0.23 | -0.1 |
| *Q2* | 22 | -0.24 | -0.32 | -0.36 | | -0.51* | -0.55** |

* *p* ≤.05; ** *p* ≤.01.

Question 1: How would you rate your / the person with autism’s QoL?

Question 2: How satisfied are you with your / the person with autism’s health?

**Supplementary Table 6. Correlations between QoL scores and adult factors**

| **Domain**  **r** |  | **IQ^1^** | **Correlations**  **Adult ADI-R algorithm^1^ Mental health rating^2^** | | | | | | | | | **Social rating** | | **Age** |  |
| --- | --- | --- | --- | --- | --- | --- | --- | --- | --- | --- | --- | --- | --- | --- | --- |
| **Informant** |  |  | **RSI** | **Communication** | | | **RRBI** | **Total** | | |  |  | |  |  |
| Physical | 47 | .13 | -.25 | -.10 | | | -.06 | -.20 | | | .03 | -.23 | | -.34* |  |
| Psychological | 45 | .25 | -.25 | .05 | | | -.25 | -.19 | | | .07 | -.25 | | -.09 |  |
| Social | 45 | -.04 | -.04 | -.20 | | | -.16 | -.16 | | | -.02 | -.04 | | -.17 |  |
| Environment | 49 | -.10 | .08 | .10 | | | -.10 | .08 | | | -.06 | .09 | | .09 |  |
| Q1 | 49 | .01 | .08 | -.16 | | | .02 | -.16 | | | -.09 | .24 | | -.26 |  |
| Q2 | 50 | .07 | .27 | ..08 | | | .18 | .19 | | | -.18 | -.23 | | -.33 |  |
| **Self-report** |  |  |  |  | | |  |  | | |  |  | |  |  |
| Physical | 22 | -.17 | .15 | .17 | | | -.22 | .09 | | | -.11 | .19 | | -.24 |  |
| Psychological | 22 | -.31 | .07 | .05 | | | -.28 | -.02 | | | -.05 | .20 | | -.08 |  |
| Social | 22 | -.56* | .21 | .13 | | | .01 | .17 | | | .27 | .57** | | .03 |  |
| Environment | 22 | .04 | .08 | .06 | | | -.05 | .06 | | | .20 | .17 | | -.09 |  |
| Q1 | 22 | -.19 | .02 | -.06 | | | -.23 | -.13 | | | -.24 | .12 | | .07 |  |
| Q2 | 22 | .05 | .20 | -.13 | | | -.59** | -.09 | | | -.19 | .12 | | .07 |  |
|  |  |  |  | |  |  | | |  |  | | |  | |  |

^1^ One participant who completed the self-report WHOQOL-BREF was missing ADI and IQ scores at follow-up ^2^ Two participants who had informant data were missing a mental health outcome score. **p* ≤.05; ** *p* ≤.01.

**Supplementary Table 7. WHOQOL-BREF : Informant ratings for individuals showing a decline in IQ over time.**

|  |  | | |  | |  | | |  |  |  |  |
| --- | --- | --- | --- | --- | --- | --- | --- | --- | --- | --- | --- | --- |
| **QoL Domain** | | | **No decline**  **(n=39)** | | **Decline**  **(n=13)** | | |  |  |  |  |  |
| Physical | | | ***Mean (sd)***  74.7 (10.8) | | ***Mean (sd)***  64.9 (19.2) | | | **t**  2.16* | | | d  .62 | |
| Psychological | | | 65.3 (13.8) | | 55.7 (7.6) | | | 2.87* | | | .69 | |
| Social | | | 55.8 (14.8) | | 57.1 (15.2) | | | 0.24 | | | .09 | |
| Environment | | 73.9 (9.2) | | | 75.8 (13.4) | | | 0.45 | | | .21 | |
| Q1 | | 4.3 (0.5) | | | 4.2 (1.1) | | | 0.36 | | | .12 | |
| Q2 | | 3.4 (0.8) | | | 3.3 (0.7) | | 0.27 | | | .13 | |  |

**Supplementary Table 8. WHOQOL-BREF scores for participants: (a) in the current study (b) Hong et al., autism sample; (c) Skevington & McCrate population sample.**

|  |  |  |  |  |  |
| --- | --- | --- | --- | --- | --- |
|  | ***Current study*** |  | ***Hong et al., 2016*** |  | ***Skevington & McCrate, 2012). Population norms*** |
| **WHOQOL-BREF domain** | ***Informant^1^***  ***(n=45-50)*** | ***Autism***  ***Participants^2^***  ***(n = 20–22)*** | ***Informant***  ***(n=60)*** | ***Autism participants***  ***(n=60)*** | ***Self-report***  ***(n* =1324–1328)** |
| Physical | *Mean (sd)*  72.4 (13.7) | *Mean (sd)*  81.1 (9.9) | *Mean (sd)*  72.1 (17.6) | *Mean (sd)*  73.3 (15.7) | *Mean (sd)*  76.5 (16.2) |
| Psychological | 63.2 (13,2) | 72.1 (15.8) | 66.5 (16.1) | 69.5 (18.8) | 67.7 (15.6) |
| Social | 56.1 (14,7) | 69.5 (23.3) | 60.2 (24.1) | 71.2 (26.9) | 70.5 (20.7) |
| Environment | 74.4 (10.3) | 76.6 (11.0) | 74.6 (18.1) | 77.4 (15.6 | 68.2 (13.8) |

In the present sample the proportions scoring within at least one standard deviation of population norms were:

Informant ratings: Physical 89%; Psychological 78%; Social 80%; Environment 98%.

Self-ratings: Physical 100%; Psychological 91%; Social 91%%; Environment 100%.
